# The protective effect of L-glutamine against acute Cantharidin-induced Cardiotoxicity in the mice

**DOI:** 10.1186/s40360-020-00449-8

**Published:** 2020-10-01

**Authors:** Haozhen Shao, Lei Dong, Yanyan Feng, Chunhui Wang, Hongxuan Tong

**Affiliations:** 1 Institute of Basic Theory for Chinese Medicine, China Academy of Chinese Medical Sciences, Beijing, 100700 China; 2 School of Life Sciences, Beijing University of Chinese Medicine, Beijing, 10029 China; 3 Fangshan Hospital of Beijing University of Chinese Medicine, Beijing, 102400 China

**Keywords:** Cantharidin, L-glutamine, Cardiotoxicity, Poisonousness reduces, Mitochondria

## Abstract

**Background:**

Cantharidin (CTD) is a compound which have the potential to be exploited as an antitumor drug, and it has been demonstrated antitumor effects in a variety of cancers. However, the use is limited due to its severe toxicity. It has reported that it can induce fatal cardiac arrhythmias. Fortunately, we found that L-glutamine can alleviate cardiac toxicity caused by cantharidin in mice.

**Methods:**

To investigate the protective effect of L-glutamine, we used a high dose of cantharidin in mice to create a model of cardiotoxicity. In the experimental mice, glutamine was given orally half an hour before they were administrated with cantharidin. The mice of control group were intraperitoneally injected with DMSO solution. The general state of all mice, cardiac mass index, electrocardiogram change and biological markers were determined. Hematoxylin-eosin staining (HE staining) of heart tissue was carried out in each group to reflect the protective effect of glutamine. To investigate the mechanisms underlying the injury and cardio-protection, multiple oxidative stress indexes were determined and succinate dehydrogenase activity was evaluated.

**Result:**

The results showed that L-glutamine (Gln) pretreatment reduced weight loss and mortality. It also decreased the biological markers (*p* < 0.05), improved electrocardiogram and histological changes that CTD induced cardiotoxicity in mice. Subsequently, the group pretreated with L-glutamine before CTD treatment increases in MDA but decreases in SOD and GSH, in comparison to the group treated with CTD alone. Besides, succinate dehydrogenase activity also was improved when L-glutamine was administrated before cantharidin compared to cantharidin.

**Conclusions:**

This study provided evidence that L-glutamine could protect cardiac cells against the acute cantharidin-induced cardiotoxicity and the protective mechanism of glutamine may be related to the myocardial cell membrane or the tricarboxylic acid cycle in the mitochondria.

## Background

Cantharidin (CTD) is extracted from blister beetle as one of the terpenoid compound used in treating cancers [1]. From latest previous studies, it found that cantharidin showed potential anticancer activities in wide variety of tumor, such as lung cancer [2], gastric cancer [3], pancreatic cancer [4–6], epidermoid carcinoma [7]. In terms of the mechanism, cantharidin can induce the apoptosis of cancer cells by suppressing the activity of protein phosphatase 2A [4, 8]. Meanwhile, the side effects of cantharidin also should be understood in more detail and attenuated before it can be better and safely used for the management of cancers.

It has many reports suggested that cantharidin can cause toxicity in many organs, such as hepatotoxicity [9], nephrotoxicity [10], and urinary system damage [11]. Most importantly, Rabkin SW has reported that cantharidin shows cardiotoxic properties because it can induce fatal cardiac arrhythmias. Therefore, as a model of cardiac arrhythmias, it was a tool to research the curative effect of antiarrhythmic drugs [12]. Accordingly, we have created a mouse poisoning model to screen for drugs that reduce the toxicity of cantharidin. Fortunately, we found that glutamine could relieve its side effects, to some extent.

In mammalian heart cells, L-glutamine (Gln) is one of the major intracellular free amino acids [13]. Gln plays an essential part for the function of biologic organs and tissues, such as heart [14]. It can also be used as a source of energy. Mao Y et al. found that Gln could improve cardiac function against acute myocardial infarction and regulate the metabolism of endothelial nitric oxide in the heart [15, 16]. It was also reported that L-glutamine has a protective effect based on the myocardial antioxidant defense system, where oxidative stress causes proteins [17], DNA [18] and lipids [19] damage, subsequently cardiac damage.

The efficacy of oral glutamine was established in doxorubicin-induced cardiotoxicity [20]. Hence, we wonder whether glutamine can also protect against CTD-induced heart damage in mice. In our study, we investigated the mechanism of Gln by testing the value of SOD and MDA related to the antioxidant defense system and by evaluating the activity of succinate dehydrogenase in the TCA cycle. Therefore, we hypothesized that disruption of the myocardial mitochondria is the cause of damage produced by cantharidin on the heart, and that L-glutamines’ antioxidant properties may be the reason for its ability, to a certain extent, to protect myocardial mitochondria.

## Methods

### Drugs and chemicals

**Fig. 1:**
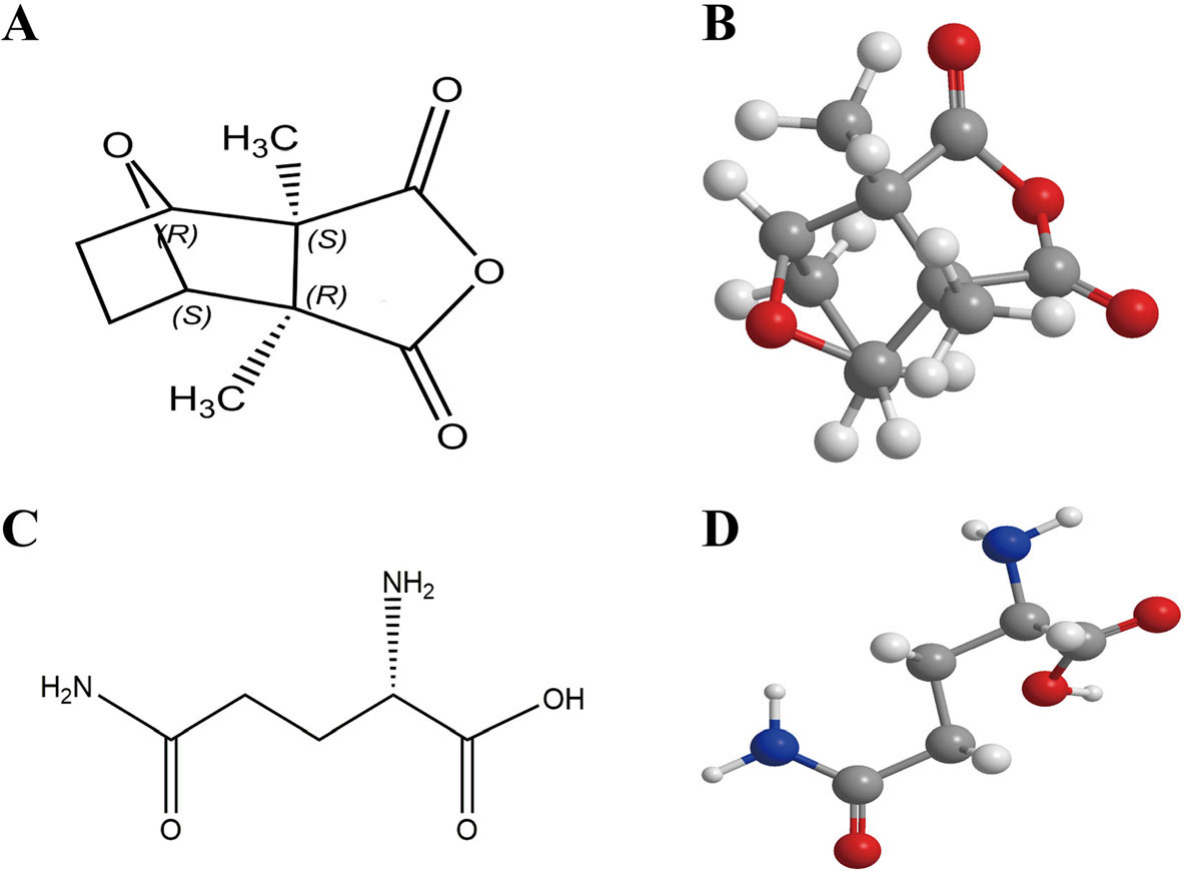
The molecular formula of cantharidin and L-glutamine. **a**: Two-dimensional chemical structure of cantharidin; **b**: Three-dimensional chemical structure of cantharidin; **c**: Two-dimensional chemical structure of L-glutamine; **d**: Three-dimensional chemical structure of L-glutamine

Cantharidin (purity ≥98%, detail molecular formula in Fig. 1a and b) was purchased from Chengdu Must Bio-Technology CO., Ltd. L-glutamine was purchased from Beijing Topbio Science & Technology CO., Ltd. (purity ≥98%, detail molecular formula in Fig. 1c and d).

### Animals and treatment

All balb/c mice were females aged 6 weeks, weighed 18 ± 2 g, and were obtained from SPF (Beijing) Biotechnology Co., Ltd. All of the mice were raised at a temperature of 25 ± 2 °C and a humidity of 60 ± 1% under a 12-h dark/light cycle with standard food and clean water in an animal facility. Mice were euthanized by cervical dislocation after completion of the experiment.

To clearly state the cardiac toxicity of cantharidin and the regulation of glutamine, we assessed the low dose (2 mg/kg) and high does (3 mg/kg) combined with L-glutamine. First 20 mice were assigned to the 4 groups randomly: (1) Control group: mice were intraperitoneally injected with a 200 μL DMSO solution. (2) CTD group: mice were treated with equal volumes of 2 mg/kg cantharidin-DMSO solution. (3) Gln 1 + 2 mg/kg CTD group: mice were pre-treated with 1 g/kg glutamine orally before being injected with 2 mg/kg cantharidin-DMSO solution. (4) Gln 2 + 2 mg/kg CTD group: mice were pre-treated with 2 g/kg glutamine orally before being injected with 2 mg/kg cantharidin-DMSO solution. All Intervention is given every 2 days in four group in 5 days.

Then, we assess the survivorship curve for 3 mg/kg cantharidin in which there were 36 mice were randomly assigned to 3 groups: (1) CTD group: mice were treated with equal volumes of 3 mg/kg cantharidin-DMSO solution. (2) Gln 1 + CTD group: mice were pre-treated with 1 g/kg glutamine orally before being injected with 3 mg/kg cantharidin-DMSO solution. (3) Gln 2 + CTD group: mice were pre-treated with 2 g/kg glutamine orally before being injected with 3 mg/kg cantharidin-DMSO solution. All Intervention is given every 2 days in three group in 5 days.

In order to assess the detail cardiotoxicity for cantharidin and the protection of L-glutamine, 24 mice were assigned to the 4 groups randomly: (1) Ctrl group: mice were intraperitoneally injected with a 200 μL DMSO solution. (2) CTD group: mice were treated with equal volumes of 3 mg/kg cantharidin-DMSO solution. (3) Gln 1 + CTD group: mice were pre-treated with 1 g/kg glutamine orally before being injected with 3 mg/kg cantharidin-DMSO solution. (4) Gln 2 + CTD group: mice were pre-treated with 2 g/kg glutamine orally before being injected with 3 mg/kg cantharidin-DMSO solution. The administration was only one time and 4 h after administration we would collect the biological tissues from mice in order to test Biological markers of cardiotoxicity, Histopathological studies, enzymatic biomarkers of oxidative stress and Succinate dehydrogenase staining.

### ECG test

Twenty mice were assigned to the 4 groups randomly: (1) Control group: mice were intraperitoneally injected with a 200 μL DMSO solution. (2) CTD group: mice were treated with equal volumes of 3 mg/kg cantharidin-DMSO solution. (3) Gln 1 + CTD group: mice were pre-treated with 1 g/kg glutamine before being injected with 3 mg/kg cantharidin-DMSO solution. (4) Gln 2 + CTD group: mice were pre-treated with 2 g/kg glutamine before being injected with 3 mg/kg cantharidin-DMSO solution. After 3 h of administration, 45 mg/kg sodium pentobarbital was injected intraperitoneally to anesthetize mice. After the anesthesia, mice were fixed to the mouse plate with tape by supine position. Spray the mouse hair with an alcohol spray can and disinfect it. Use a curved scissors to shave the hair off the mouse’s chest (or use a shaver). Connect the leads of the electrocardiograph (Cardimax FX-7202) to the limbs and the heart, as required. Connect the power and print the ECG drawing after the heart rate is basically stable. Scan ECG drawings and analyze changes in each band.

### Biological markers of cardiotoxicity

After electrocardiographic assessment, blood was collected from retro-orbital plexus under mild anesthesia. Mice were sacrificed and the hearts were collected immediately. The fresh heart was divided into three parts: one part of the heart was fixed in the 4% formalin for histopathological examination; the other parts of the heart were immediately embedded with a frozen biopsy agent; and the remaining parts were collected for biochemical analysis.

Serum was collected through centrifugation at 3500 rpm (10 min, 4 °C). Serum levels of creatine kinase (CK), creatine kinase-MB isoenzyme (CK-MB), lactate dehydrogenase (LDH) enzymes, and aspartate aminotransferase (AST) were tested by automated chemistry analyzer (CX4 Pro, Beckman Coulter, USA) using reagent kits.

### Histopathological studies

The heart was sliced into small pieces and preserved for 24 h in 4% formalin. Specimens were sliced into sections 4 mm thick. The specimen was made transparent by xylene, and subsequently mounted by neutral resin, then stained by hematoxyline and eosin.

### Effect of oxidative stress on enzymatic biomarkers

The one part of heart tissues was homogenized in chilled homogenizing buffer by a tissue homogenizer and centrifuged at 3500 rpm.(10 min, 4 °C). The SOD activity was estimated in the serum and myocardium using the Marklund & Marklund method [21]. GSH content was estimated by the Subramaniam method. MDA content in supernatant of the mice was tested by Slater and Sawyer method [22]. Protein concentrations were tested by the BAC protein detection kit. (Beijing Solarbio Science & Technology Co., Ltd.)

### Succinate dehydrogenase(SDH)staining

The second portion of isolated heart was embedded with a frozen biopsy agent and immediately placed in a − 80 °C environment. The frozen sections were made of Leica frozen microtome and stained using a Succinate dehydrogenase staining kit (Beijing Solarbio Science & Technology Co., Ltd.)

### Statistical analysis

The data form was the mean ± S.D. which was analyzed by IBM SPSS statistical software package (version 20). Statistical comparisons were made using Student’s t-test and one-way analysis of variance (ANOVA). *p* < 0.05 was considered as statistically significant.

## Results

### The general state of mice

**Fig. 2:**
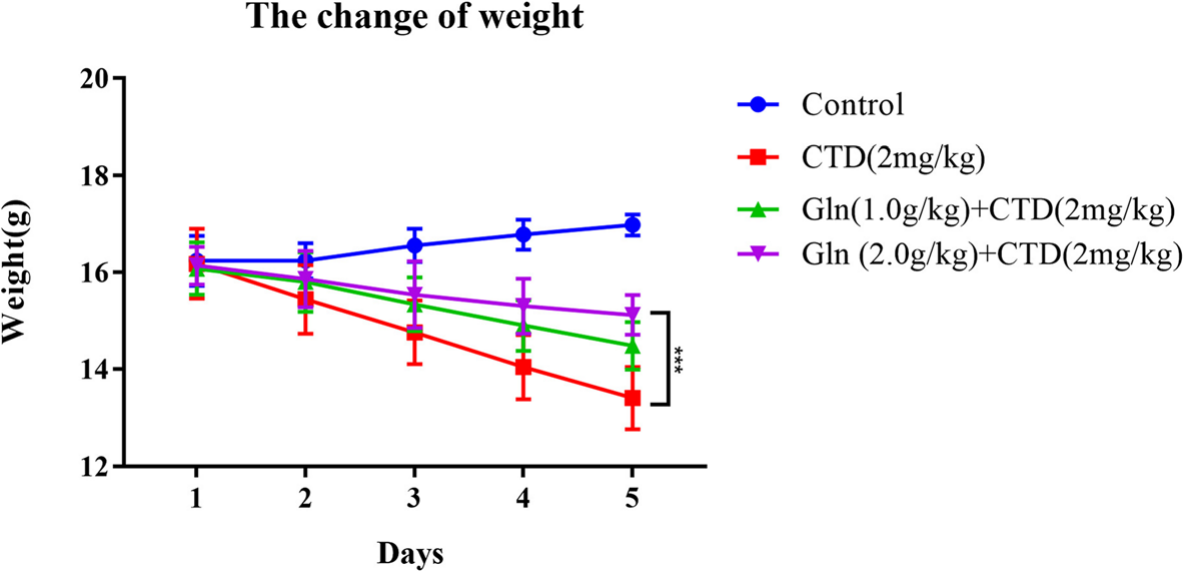
The change of weight for cantharidin combined with glutamine. *** means the group administrated with 2 mg/kg cantharidin had a statistical difference compared with the group administrated with 2 g/kg glutamine combined with 2 mg/kg cantharidin (*p* < 0.001). *N* = 5

**Fig. 3:**
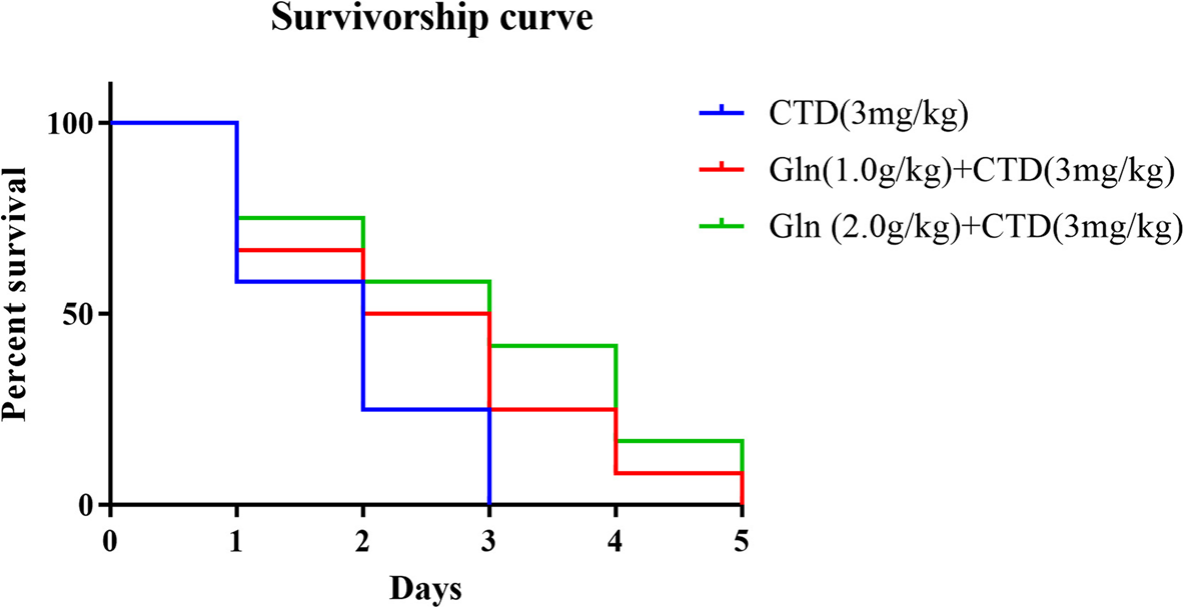
The survivorship curve for cantharidin combined with glutamine. Glutamine showed its protective effect against cantharidin-induced cardiotoxicity that the cantharidin combined with glutamine would lead to slower death compared with the single using of 3 mg/kg cantharidin. *N* = 12

First, weight change showed that all of three groups administrated with 2 mg/kg cantharidin loss their weight significantly compared with control group. Meanwhile, the groups 1 g/kg glutamine combined with 2 mg/kg cantharidin and 2 g/kg glutamine combined with 2 mg/kg cantharidin showed a good protection effect, especially using 2 g/kg glutamine which had a significant effect compared with CTD group (Fig. 2). We also assessed the survivorship curve in high dose cantharidin (3 mg/kg cantharidin) combined with glutamine, it showed that the single using of 3 mg/kg cantharidin would lead to quicker death compared with cantharidin combined with glutamine (Fig. 3).

### Cardiac mass index

**Fig. 4:**
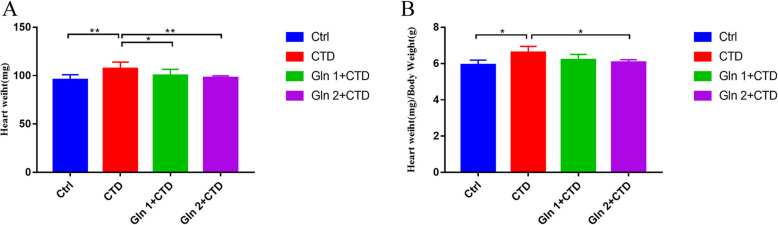
Cardiac mass index. The glutamine was able to reduce the damage induced by CTD. * means *p* < 0.05, ** means *p* < 0.01. *N* = 6

Then, we assessed the acute toxicity of CTD (3 mg/kg) which was used alone or combined with glutamine. The mouse and its heart were weighed, the ratio of heart weight to body mass (mg/g) was calculated. The groups of Gln 1 + CTD and Gln 2 + CTD showed that the increase in heart weight was lower than CTD group. The ratio of heart weight/body mass (mg/g) in the group of Gln 2 + CTD was lower than the group of CTD (Fig. 4).

### Electrocardiogram change

**Fig. 5:**
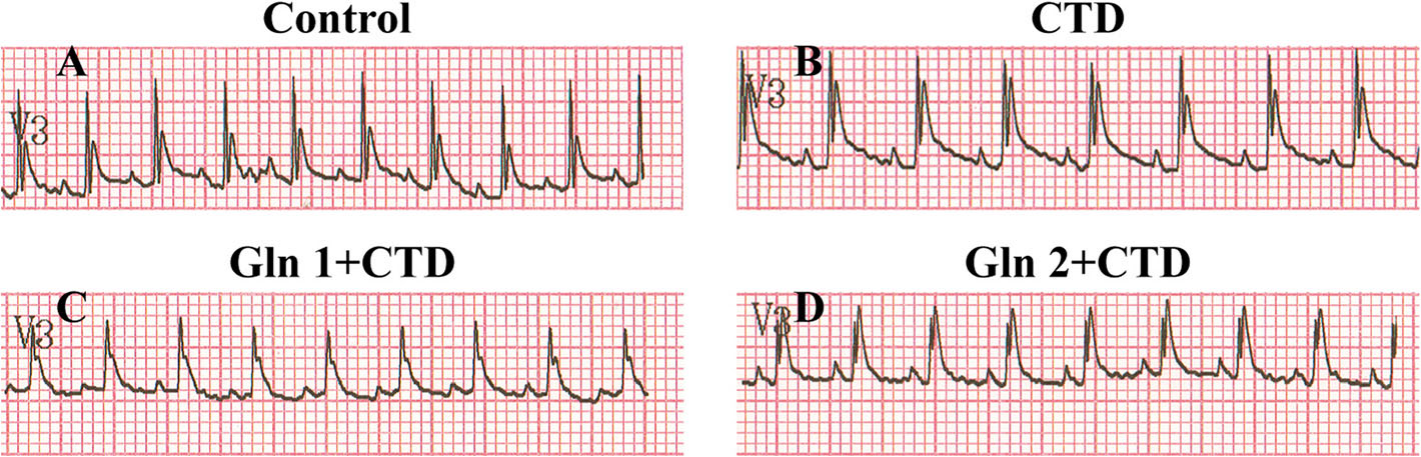
The ECG change in four groups. In the v3, The ST segment in CTD group was elevated higher than that of any other three groups, and CTD caused myocardial damage which could alleviate by glutamine. *N* = 6

From the ECG of different groups, we found the group administrated with 3 mg/kg cantharidin that the ST segment was higher than that of control group. Meanwhile, the ST-segment elevation of two groups, respectively administrated with 1 g/kg and 2 g/kg glutamine before giving cantharidin, were less than the group administrated with 3 mg/kg cantharidin. The myocardial damage caused by cantharidin alleviated by glutamine (Fig. 5) .

### Effect on cardiac biomarkers

**Fig. 6:**
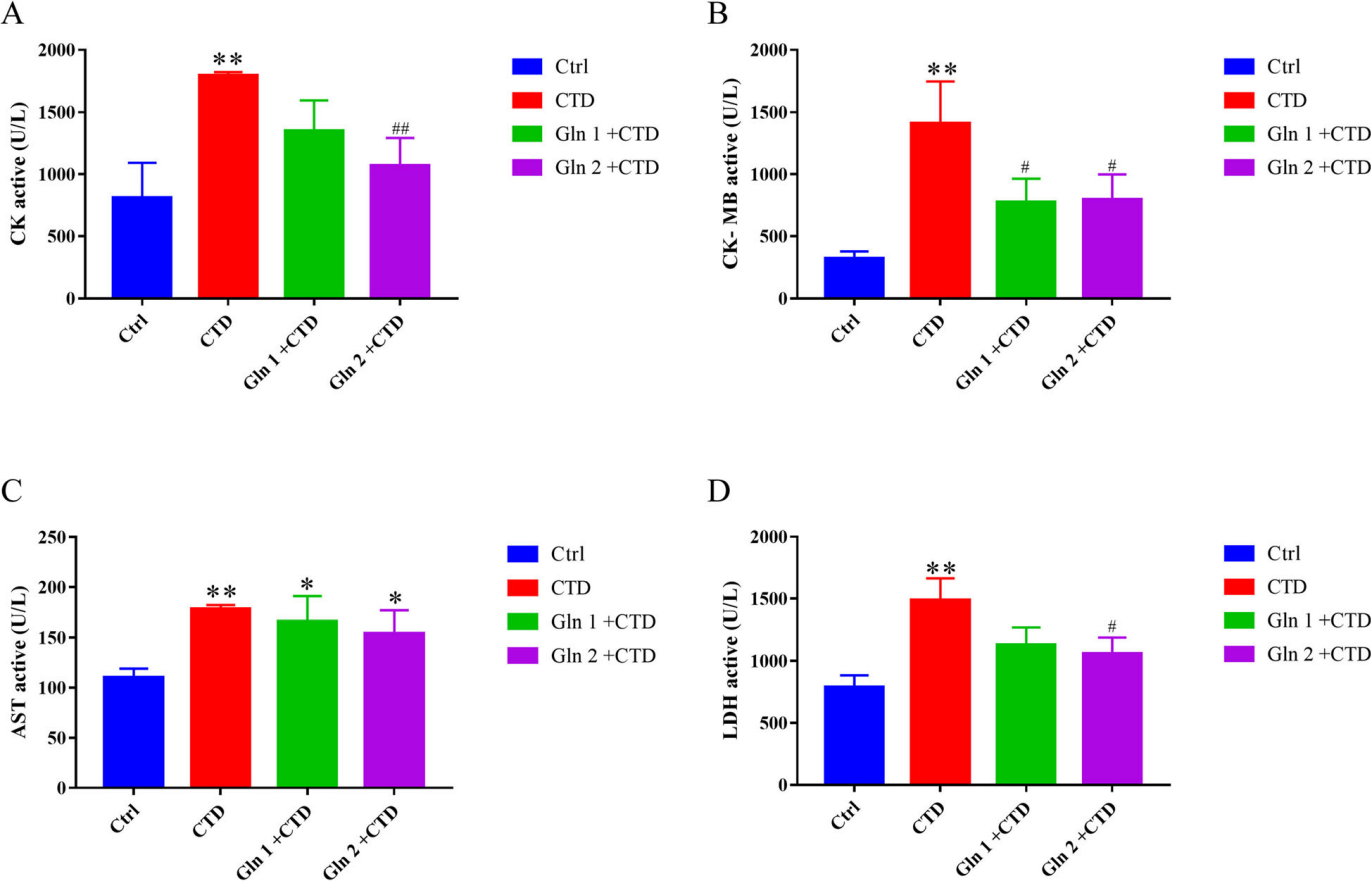
Results showing biological markers. The glutamine was able to partly decrease the CK, CK-MB, AST, LDH induced by CTD. *p < 0.05, **p < 0.01, compared with control animals. ^#^*p* < 0.05, ^##^*p* < 0.01, compared with cantharidin animals. *N* = 6

It is well known that cardiac enzymes are significant diagnostic biomarkers of myocardial damage. In the present study, CTD increases the levels of enzymes such as CK (*p* < 0.01), CK-MB (*p* < 0.01), AST (*p* < 0.01), and LDH (*p* < 0.01) significantly compared with the control group. While, pretreatment with Gln decreased the indices of enzymes which were increased by CTD (Fig. 6).

### Histopathology of mice hearts

**Fig. 7:**
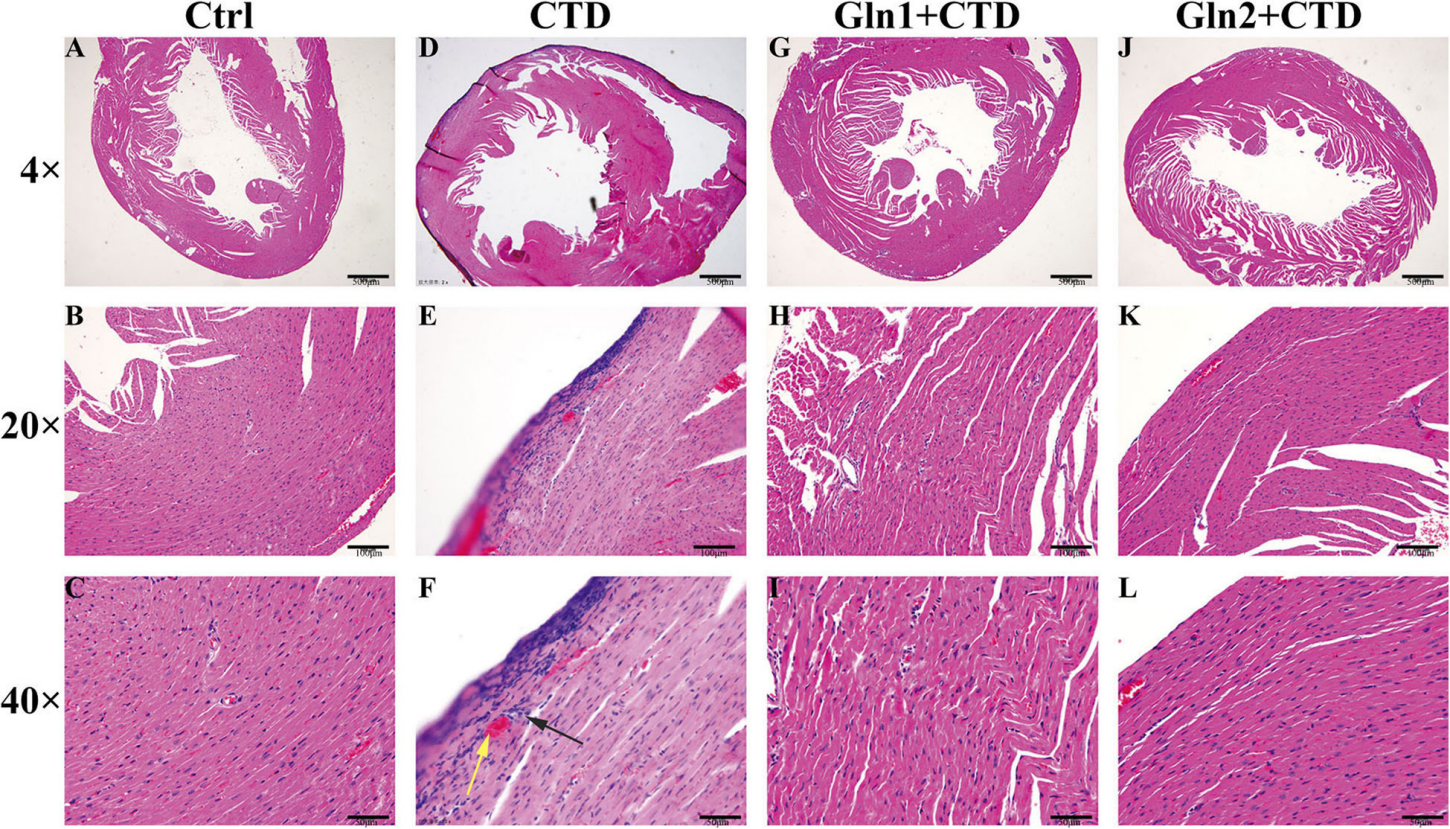
Histopathology of mice hearts by hematoxyline and eosinophil staining. Vertical rows represent the control, CTD,L-glutamine 1 mg·kg-1 + CTD and L-glutamine 2 mg·kg-1 + CTD group mice heart, respenctly. Horizontal row represent the same tissue with different scale bar as 500 μm, 100 μm and 50 μm, respectively. As the image of CTD group (Fig. 7f), yellow arrow represents myocardial interstitial hyperemia, and black arrow represents inflammation

Figure 7 showed histopathological change by HE staining for heart. The histopathological change in controlled mice showed absence of myocardial inflammation indicating normal architecture of the heart. Compared with the control group, the CTD 3 mg·kg-1 group mice showed inflamations, myocardial interstitial hyperemia (Fig. 7f). It confirms that CTD has caused damage in the heart. Pretreatment of L-glutamine reduced myocardial inflammation compared with single use of CTD (Fig. 7i and l). In conclusion, CTD caused pathological changes, while L-glutamine protected the heart tissue to some degree.

### Effect on oxidative stress parameters

**Fig. 8:**
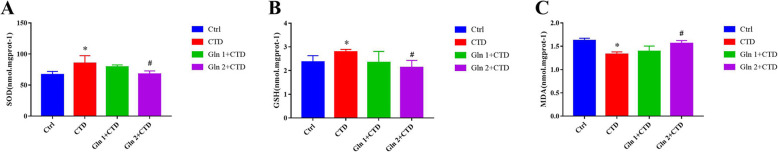
The different oxidative stress indexes of mice. *p < 0.05 compared with control animals. # means *p* < 0.05 compared with CTD animals. *N* = 6

Compared with the ctrl group, the value of SOD and GSH of the CTD group increased significantly (*p* < 0.05). Moreover, MDA reduced significantly (*p* < 0.05) (Table 1 and Fig. 8). It means that the use of CTD resulted in oxidative stress in the early stage of heart damage in mice. However, with enough volume of glutamine pretreatment in mice, the concentration of SOD, GSH, and MDA has less influence compared to the mice treated with CTD alone.

**Table 1:** Results showed oxidative stress indexes of mice. The glutamine was able to partly decrease oxidative stress parameters induced by CTD

	Ctrl	CTD	Gln 1 + CTD	Gln 2 + CTD
SOD (nmol·mgprot^−1^)	66.80 ± 5.28	85.36 ± 11.94*	79.02 ± 3.42	67.78 ± 4.97#
GSH (nmol·mgprot^−1^)	2.36 ± 0.27	2.79 ± 0.10*	2.34 ± 0.47	2.13 ± 0.30#
MDA (nmol·mgprot^−1^)	1.62 ± 0.05	1.33 ± 0.05*	1.39 ± 0.11	1.56 ± 0.06#

* means *p* < 0.05, compared with control animals. # means *p* < 0.05, compared with CTD animals. *N* = 6

### Succinate dehydrogenase staining (SDH) in frozen sections

**Fig. 9:**
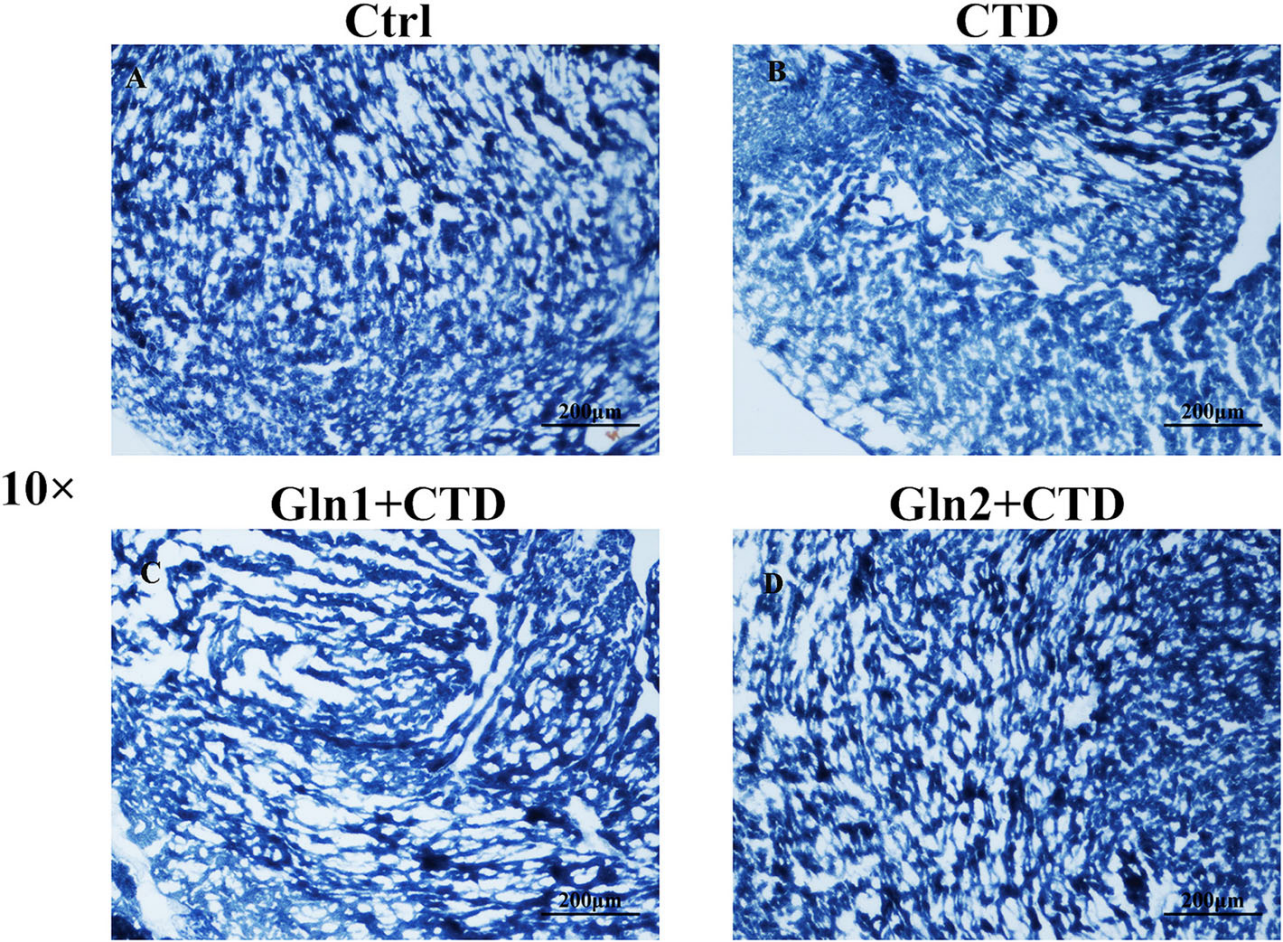
Succinate dehydrogenase staining of mice heart tissue. Scale bar is 200 μm. Ctrl, controls; CTD, cantharidin; Gln, glutamine. The heart tissue of CTD treated mice showed large areas of superficial dyeing, the blue stained color was lighter compared with the control group mice (Fig. 9b). While, the group of glutamine pretreated mice showed a deeper staining compared with the CTD group mice (Fig. 9c and d)

Figure 9 depicts the SDH staining results of the following groups of mice: the control group mice, CTD treated mice, mice that were pretreated with 1 g/kg Gln, and mice that were pretreated with 2 g/kg Gln. As we can see in Fig. 9, the heart tissue of CTD treated mice showed large areas of superficial dyeing; the blue stained color was lighter compared with the control group mice. While, the group of glutamine pretreated mice showed a deeper staining compared with the CTD group mice. This suggests that the activity of succinate dehydrogenase in CTD treated mice was lower compared with the control group and the activity of succinate dehydrogenase was recovered after administration of glutamine.

## Discussion

In this study, we showed the cardiac toxicity induced by cantharidin, and showed that glutamine is protective. For this reason, we have made a brief explanation of the discovery in terms of biochemical indicators and H&E staining.

From the mentioned results, the parameter of biomarker enzymes (CK, CK-MB, AST and LDH) in plasma increased in CTD group compared to the control mice. These biomarker enzymes reflect a non-specific aberration for the functional and structural completeness of myocardial membranes as a response to CTD damage. Histopathological studies revealed an inflammatory infiltrate in the outer ventricular membrane in CTD treated mice. There was a greater space between the cardiomyocytes, as compared to the control mice. It is consistent with the characteristics of cantharidin damaging tissues and cells reported by Fillmore K. et [23]. The disruption of the membrane structure of cardiac myocytes was further confirmed in our experiment.

Meanwhile, prior prescription of L-glutamine could decrease the release of cytosolic enzymes into circulation. L-glutamine pretreated animals showed no apparent abnormalities of histopathology compared with the control group mice, that demonstrated the protective effect on the heart cells; Yan et al. also reported that L-glutamine seems to have a protective effect after myocardial damage [24].

To further investigate the potential mechanism of the toxicity caused by CTD in the mice heart. We have determined the enzymatic biomarkers of oxidative stress, such as SOD, GSH and MDA, and stained succinate dehydrogenase, which can be used to manifest the active of mitochondrial in cardiac myocyte. As a vital defense enzyme, SOD can catalyze the dismutation of superoxide radicals. Glutathione (GSH) is able to prevent the damage of important cellular components by reactive oxygen species [25]. As the oxidative degradation product, the level of MDA is an indicator of lipid peroxidation origin from cell-membrane lipids [26]. In the present study, we found that the mice treated with cantharidin resulted in a transient elevation of SOD and GSH. Furthermore, the amount of MDA was significantly different from the mice in the control group. There are two hypotheses to explain the phenomenon.1) In the previous report, CTD can efficiently inhibit the activity of mammalian protein phosphatase 2A (PP2A) [27]. Tsung-I Chen et al. found that ROS generation increases PP2A activation, while its activation can be inhibited by the cantharidin and SOD [28]. It suggested that cantharidin may inhibit PP2A activation by increasing SOD level. 2) In the general state, the tissue would increase the SOD and GSH level to protect cells from ROS damage in a short time, but ultimately reduced SOD and GSH level because of the continued consumption [29, 30]. Considered that we took blood in an early time, it is possible. In both cases, it implies ROS response was induced in the early stages of damage in CTD-treated mice heart.

In addition, succinate dehydrogenase is the only enzyme which participates in both the electron transport chain and the citric acid cycle [31]. Chouchani showed selective accumulation of intermediate succinate in citric acid cycle is a common metabolic characteristic of ischaemia which is responsible for mitochondrial ROS production during reperfusion in a range of organs and tissues [32]. In our experiments, succinic dehydrogenase staining was lighter in the CTD group, meaning its activity decreased. In the earlier study of cantharidin, it was reported that cantharidin possibly affects the cell membrane, for example via some subcellular particles [23]. According to our data, CTD really destroys cells or mitochondria, which increases oxidative stress products and reduces mitochondrial marker enzymes. We speculate that the destruction of the cell membrane or mitochondrial membrane structure, induced by CTD, prompts the change of various makers.

As principal energy source, Glutamine was used to formate mitochondrial ATP in oxidative metabolism of mitochondria which drive the contraction of heart [33, 34]. It has been reported that sudden elimination of Gln could lead to a sharp decline of mitochondrial respiration [35]. As an enzyme complex, succinate dehydrogenase exists in the inner mitochondrial membrane of heart cells. The activity of succinate dehydrogenase in the groups of pretreated with high dose glutamine was almost the same as that of the control group mice. The protective effect may be due to the mechanism that the catalysis of glutamine into glutamate provides the urgent needed substrate, utilized as energy production in the citric acid cycle, to counteract membrane disintegration induced by cantharidin in the myocardium referred to Dumaswala’ study [36]. From our results, we found that 2 mg·ml^− 1^ L-glutamine pretreated mice showed no significant difference with the control animals on SOD, GSH and MDA parameters. It reminded us that glutamine protective role in heart tissue may be also related to the ROS response. The accumulation of succinate will be re-oxidized rapidly by succinate dehydrogenase after damage which will lead to generation of excess ROS by reverse electron transport in mitochondrial complex I. As to glutamine, it may protect several protein complexes in the mitochondria.

## Conclusion

Cantharidin showed certain anticancer activities in a wide variety of tumors in vivo and vitro, while it can cause toxicity in many organs especially in the heart. In our animal experiment, as one of the major intracellular free amino acids, L-glutamine could alleviate cardiac toxicity that caused by cantharidin in a spectrum of experiments, including ECG test, myocardial enzyme, histopathology and so on.

## Data Availability

The relevant data to work have been included in the paper. However, the supplementary data may be shared on request to the main contributing authors on request.

## Abbreviations

CTD: Cantharidin
Gln: L-glutamine
SOD: Superoxide dismutase
GSH: Cardiac glutathione
MDA: Malondialdehyde
ECG test: Electrocardiogram test
CK: Creatine kinase
CK-MB: Creatine kinase-MB isoenzyme
AST: Aspartate aminotransferase
LDH: Lactate dehydrogenase enzymes
